# Comparison of systemic trimethoprim-sulfadimethoxine treatment and intrauterine ozone application as possible therapies for bacterial endometritis in equine practice

**DOI:** 10.3389/fvets.2023.1102149

**Published:** 2023-01-27

**Authors:** Martin Köhne, Lisa Hofbauer, Denny Böttcher, Anna Tönissen, Anna Hegger, Alexandra Görgens, Reiner Ulrich, Harald Sieme

**Affiliations:** ^1^Unit for Reproductive Medicine – Clinic for Horses, University of Veterinary Medicine, Foundation, Hannover, Germany; ^2^Clinic for Horses Mühlen, Steinfeld (Oldenburg), Germany; ^3^Institute of Veterinary Pathology, Faculty of Veterinary Medicine, Leipzig University, Leipzig, Germany

**Keywords:** endometritis, ozone therapy, antibiotic treatment, non-traditional treatment, horse

## Abstract

Bacterial endometritis is one of the major problems in equine reproduction and usually treated with antimicrobial drugs. The study aimed to compare the effects of intrauterine ozone application and systemic antibiotic treatment (trimethoprim-sulfadimethoxine) on intrauterine bacterial growth and possible side effects on the endometrium in a clinical setting. Mares (*n* = 30) with signs of endometritis (positive uterine bacterial culture and cytological findings) were assigned randomly to different treatments: intrauterine insufflation of an ozone-air-mix (240 ml, 80 μg ozone/ml) twice at a 48 h-interval (Ozone; *n* = 10), systemic antibiotic therapy with trimethoprim-sulfadimethoxine (30 mg/kg, p.o., twice daily) for 5 days (TMS; *n* = 10), or intrauterine insufflation of air (240 ml, sterile-filtered) twice at a 48 h-interval (air; *n* = 10). Endometrial biopsy for histological examination was obtained before the treatment. Histological examination revealed no differences among groups. A control examination, including transrectal ultrasound, bacterial culture, cytological evaluation, and biopsy, was performed 7 days after the last treatment. Overall bacterial growth was reduced in every group after the treatment (*p* < 0.05), irrespective of the therapy [Ozone: 4/9 (positive culture after treatment/number of mares), TMS: 3/10 and Air: 6/10; *p* > 0.05]. However, Ozone and TMS (*p* < 0.05) were more effective in reducing growth of gram-negative bacteria as compared to Air (*p* > 0.05). No effects on the number of polymorphonuclear granulocytes (cytology) were observed (*p* > 0.05). In conclusion, trimethoprim-sulfadimethoxine and intrauterine ozone insufflation are safe treatment options for bacterial endometritis in mares but the efficacy of both treatments in reducing bacterial growth did not result in a complete absence of intrauterine bacterial growth.

## 1. Introduction

Chronic infectious endometritis is one of the major causes for subfertility in mares and observed in 25%−60% of barren mares (1–3). Since the causative pathogens are predominantly bacteria (e.g., β-hemolytic streptococci and *Escherichia coli*) (4, 5), the traditional therapy usually involves antibiotics (6), that are either administered locally (7) or systemically (8–11). According to the OIE guidelines for responsible and prudent use of antimicrobial agents in veterinary medicine (12), antibiotics should only be administered when necessary, after a proper examination of the animal and based on diagnostic laboratory information, where possible. The regulation (EU) 2019/6 demands a responsible use of antibiotic drugs and questions off-label usage of antibiotics [e.g., changes of administration routes (13)]. Trimethoprim-sulfadimethoxine is a broad-spectrum antibiotic, licensed for systemic treatment of infectious diseases of the equine urogenital tract in Germany and other countries. A study investigating the endometrial tissue availability of the analogous trimethoprim-sulfadiazine demonstrated that minimal inhibitory concentrations against *E. coli* and *Streptococcus equi* ssp. *zooepidemicus* were exceeded in the equine endometrial tissue (14) but clinical data is lacking.

Besides the demand for responsible use of antibiotic drugs, the regulation (EU) 2019/6 also asks for further research on alternative therapeutic methods. Information on alternative treatment options as performed in the field is often scarce, although various reports on such treatments exist (15). One of the non-traditional treatments performed is insufflation of ozone as an alternative antibacterial treatment. Ozone has bactericidal properties due to its powerful oxidizing effect, targeting the bacterial membrane glycoproteins, glycolipids as well as amino acids (16). Therefore, it has been extensively used in the food industry (17) and in dentistry (18). In veterinary medicine, positive effects of ozone treatment have not only been demonstrated for the treatment of acute clinical mastitis in cattle (19) but also for resolving different pathological conditions of the bovine reproductive tract (e.g., urovagina and retained fetal membranes) (20). Regarding possible applications of ozone in equine reproduction, effects of ozone on cooling and cryopreservation of equine semen have been evaluated recently and found to be beneficial when administered in low concentrations (21). In mares, intrauterine application of an oxygen-ozone gas mixture was able to induce endometrial angiogenesis and did not negatively affect endometrial histomorphology (22). Another study investigated the ability of ozone to disrupt biofilms and kill gram-negative equine uterine pathogens *in vitro* but did not find ozone to reliably reduce growth of gram-negative bacteria (23). Only recently, a study from Brazil demonstrated the potential of intrauterine ozone insufflation as a viable treatment option for infectious endometritis (24).

The aim of the present study was to evaluate the efficacy of intrauterine ozone administration and systemic trimethoprim-sulfadimethoxine treatment in resolving bacterial endometritis in mares in a clinical setting. Moreover, possible effects of the treatments on the endometrium were evaluated. The hypothesis of the study was that ozone as well as trimethoprim-sulfadimethoxine would be able to resolve bacterial endometritis in mares without affecting theendometrium.

## 2. Materials and methods

### 2.1. Selection of mares

After submission to the clinic for artificial insemination by the owner, mares underwent a complete clinical examination. When no abnormalities were found, a gynecological examination, consisting of rectal palpation and ultrasonography of the ovaries and the uterus, was performed. Clinical signs of endometritis (e.g., intrauterine fluid, hyperedema or vaginal discharge) were noted, but not a mandatory inclusion criterion. After the tail was wrapped, the vulva, and the perineum were cleaned, endometrial samples for microbiological and cytological assessment were obtained as described in Section 2.3. Mares with a uterine culture positive for *S. equi* ssp. *zooepidemicus, Streptococcus dysgalactia*e or undefined β-hemolytic streptococci were included into the study regardless of the result of the cytological examination. Mares with a culture positive for *E. coli* were included into the study, if cytological investigation revealed the presence of at least five inflammatory cells per 10 analyzed high power fields (HPFs) (25).

During the 2021 breeding season from February to August, the criteria were met by 28 Warmblood mares, one Shagya Arabian and one Galineers cob mare (*n* = 30), privately owned and submitted to the Clinic for Horses Mühlen, Germany for insemination. The mares were maiden (*n* = 9), foaling (*n* = 4) or barren (*n* = 17). Their age was between 4 and 26 years [mean: 12.9 ± 5.5 (mean ± SD)]. Additional information on the mares can be found in Table 1. Throughout the experiment, the mares were kept in straw-bedded boxes in a separate stable and fed oats and hay twice daily. Water was freely available and mares were moved to paddocks once daily. Experiments were performed in agreement with German animal welfare legislation and approved by the Lower Saxony State Office for Consumer Protection and Food Safety (33.8-42502-04-20/3565).

**Table 1:** Information on individual mare characteristics, categorization of mares before treatment according to Kenney and Doig (26) and Schoon et al. (27) and signs of endometritis before and after treatment as determined by endometrial biopsy.

**Mare (no.)**	**Age (years)**	**Reproductive status of mare**	**Treatment group**	**Categorization**	**Signs of endometritis before treatment (biopsy)**	**Signs of endometritis after treatment (biopsy)**
1	20	Barren	Ozone	III	Mild	Moderate
2	14	Maiden	Ozone	III	Moderate	Moderate
3	12	Barren	Ozone	III	Moderate	Mild
4	6	Barren	Ozone	IIa	Mild	Mild
5	17	Maiden	Ozone	III	Mild	Mild
6	6	Barren	Ozone	III	Mild	Mild
7	10	Barren	Ozone	IIb	Mild	Mild
8	15	Maiden	Ozone	IIb	Mild	Moderate
9	14	Foaling	Ozone	IIb	Mild	Mild
10	4	Maiden	TMS	IIa	None	Mild
11	8	Maiden	TMS	III	Mild	Moderate
12	4	Maiden	TMS	IIb	Mild	Mild
13	18	Barren	TMS	III	Mild	Mild
14	26	Barren	TMS	III	Severe	Moderate
15	10	Maiden	TMS	III	Moderate	Mild
16	12	Barren	TMS	IIb	None	Moderate
17	16	Barren	TMS	III	Mild	Mild
18	13	Foaling	TMS	IIb	None	Mild
19	8	Barren	TMS	III	Moderate	Mild
20	16	Barren	Air	III	Mild	Mild
21	5	Barren	Air	IIb	Mild	None
22	9	Foaling	Air	III	Mild	Mild
23	11	Foaling	Air	III	Moderate	Mild
24	14	Barren	Air	III	Moderate	Moderate
25	19	Barren	Air	III	Moderate	Moderate
26	16	Maiden	Air	III	Moderate	Moderate
27	23	Barren	Air	III	Moderate	Mild
28	12	Maiden	Air	III	Moderate	Mild
29	Unknown	Barren	Air	I	None	Moderate

### 2.2. Experimental design

**Figure 1:**
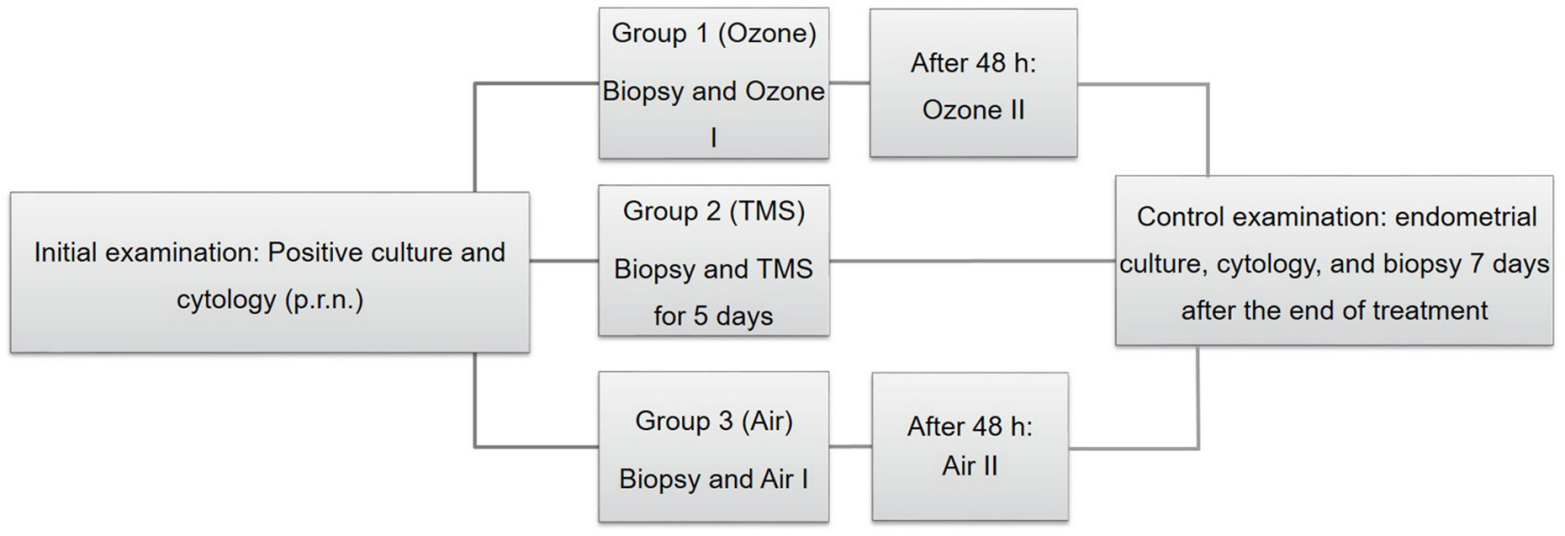
Schematic depiction of the experimental design, including the initial examination **(on the left)** treatment of groups [Group 1–3; **(center)**] and the control examination **(on the right)**. TMS, trimethoprim-sulfadimethoxine.

As soon as microbiological and cytological results were evaluated, mares were assigned randomly to three different treatment groups. Before the onset of the respective therapy, mares were examined gynecologically (incl. ultrasonography) for determination of cycle stage and presence of signs of clinical endometritis (e.g., intraluminal uterine fluid, endometrial hyperedema, vaginal discharge). Afterwards, the tail was wrapped and cleaning of the perineum and vulva was performed with a disinfectant soap once (Degraseptin, Braun, Melsungen, Germany). An endometrial biopsy was obtained transcervically regardless of the mares' cycle stage. Biopsy samples were fixed in 4% buffered formaldehyde solution and sent to the Institute of Veterinary Pathology, Faculty of Veterinary Medicine, Leipzig University, Leipzig, Germany. Immediately afterwards, mares were treated according to their experimental group (Figure 1).

In group 1 (ozone, *n* = 10), an ozone-air mixture (240 ml) was administered into the uterus with a flexible insemination pipette (67 cm, Minitüb, Tiefenbach, Germany) and two syringes (120 ml, Braun, Melsungen, Germany). Briefly, the ozone-gas mixture was produced on site with a medical-grade ozone generator (Humadent, Humares, Bruchsal, Germany) and had a concentration of 80 μg/ml. The ozone generator was equipped with a collection valve for connecting the syringes, where the syringes were filled with 120 ml ozone-air mixture within 10 s. Immediately afterwards, the filled syringe was handed to the person performing the infusion and connected to the insemination pipette, which had already been introduced into the uterus transcervically. During the inflation procedure, the responsible person ensured the correct position of the tip of the insemination pipette and occluded the cervix manually to prevent loss of gas for 2 min. No additional measures were taken to control the removal from the ozone-air mixture within the next 48 h. After 48 h, the application of the ozone-gas mixture was repeated.

Mares in group 2 (TMS, *n* = 10) received trimethoprim-sulfadimethoxine systemically (30 mg/kg, twice daily, p.o.; Trioxin, Bela-Pharm, Vechta, Germany) for 5 days in accordance with the antibiotic susceptibility test results.

In group 3 (air, *n* = 10), sterile-filtered room air was administered into the uteri according to the application of the ozone-air mixture (group 1).

Seven days after the last treatment, a control examination, including ultrasonography and collection of samples for uterine microbiological and cytological assessment as well as endometrial biopsy was performed regardless of the treatment group and cycle stage. All treatments were performed irrespective of the mares' cycle stage. Mares did not receive any other additional treatment between initial and control examination.

### 2.3. Microbiological sampling and result interpretation

During the experiment, two different methods for obtaining uterine cultures were performed. For reasons of time, the majority of mares (*n* = 28) was swabbed with a double-guarded swab (uterine culture swab, Minitüb, Tiefenbach, Germany), which was introduced manually into the uterus after initial cleaning of the vulva and perineum as described in Section 2.2. Low volume-lavage as described by Christoffersen et al. (28) was used with slight modifications in two mares (one mare in group 1 and one mare in group 3) at both samplings. Briefly, vulva and perineum were cleaned and single-use uterus flushing tube (EQUIVET, Jørgen Kruuse A/S, Langeskov, Denmark) connected to a fluid bag (250 ml, 0.9% NaCl, Braun, Melsungen, Germany) and protected by a sterile metal protection tube and a clean rectal examination glove was introduced into the vagina. At the level of the external cervical orifice, the protection tube was advanced through the rectal examination glove and inserted into the cervical canal. Then the uterus flushing tube was advanced ~10 cm into the uterine body and the uterus was flushed by gravity flow until at least 80 ml of the saline solution were recovered. Subsequently, the recovered fluid was aspirated with a syringe, filled into conical centrifugation tubes (50 ml) and centrifuged at 400 × *g* for 10 min. One resulting pellet was transferred immediately into AMIES charcoal medium and transported to an internationallyd accredited laboratory (Labor Dr. Böse, Harsum, Germany) for microbiological examination at the same day. The pellet from the second centrifugationd tube was used for cytological examination. Manually obtained swabs were also transported in AMIES charcoal medium to the laboratory within 24 h. Aerobic cultured and antibiotic susceptibility testing were performed as described by Spilker et al. (29) and according to the Clinical & Laboratory Standardsd Institute (CLSI) Guidelines. If aerobic culture revealed the presence of *S. equi* ssp. *zooepidemicus, S. dysgalactia*e or undefinedd β-hemolytic streptococci or *E. coli* (in combination with the above-mentioned cytological criteria), it was considered positive.

### 2.4. Cytological sampling and evaluation

For cytological examination, either the aspirated pellet of the low-volume lavage or superficial endometrial cells collected *via* cytobrush (Minitüb, Tiefenbach, Germany) were smeared on a prestained glass slide (Testsimplets, Waldeck, Münster, Germany), air-dried and fixed with a spray fixative (M-Fix, Merck Darmstadt, Germany) before evaluation. Cytobrush sampling was performed manually after cleaning of the perineal area and microbiology sampling as described in Section 2.2. Slides were examined by light microscopy (Olympus BX41, Olympus Deutschland, Hamburg, Germany) at 400 × magnification for presence of polymorphonuclear neutrophils (PMNs). A minimum of 200 cells and 10 HPFs were analyzed and PMNs were counted. According to Dascanio (25), < 1 PMNs per HPF were considered as normal, 1–2 PMNs/HPF were regarded as indicative for mild, 3–5 PMNs/HPF for moderate and >5 PMNs/HPF for severe endometritis.

### 2.5. Endometrial biopsy and histological examination

Endometrial biopsies were collected transcervically from the base of one uterine horn with a Ferris biopsy forceps (65 cm, Eickemeyer, Tuttlingen, Germany). The endometrial biopsies were immediately transferred into 4% buffered formaldehyde and fixed for 24–48 h. After arrival at the Institute for Veterinary Pathology (Leipzig University, Germany) the samples were automatically dehydrated in a graded series of alcohol (Donatello, Diapath, Martinengo, Italy) and embedded in paraffin (Paraplast, Engelbrecht, Edermünde, Germany). The paraffin-embedded tissue was cut into 3–4 μm thin slices using a sliding microtome (SM2010 R, Leica, Wetzlar, Germany). The sections were mounted on glass slides and dried for 15 min at 60°C in a heating chamber. Staining was performed with haematoxylin and eosin (HE). All slides were scanned with a slide scanner (Axioscan 7, Carl Zeiss Microscopy, Jena, Germany) equipped with 40 × magnifying objective (Plan-Apochromat 40 × /0.95, Carl Zeiss Microscopy, Jena, Germany) and examined histologically using ZEISS ZEN 3.4 blue edition, Carl Zeiss Microscopy GmbH, Jena, Germany. Particular interest was paid to the presence of inflammatory cells, fibrosis, desquamation of the endometrial surface epithelium and extravascular erythrocytes. Categorization of mares occurred based on the pre-treatment biopsy according to the categorization scheme by Kenney and Doig (26), modified by Schoon et al. (27).

### 2.6. Statistical analysis

Data were statistically analyzed using IBM SPSS software (IBM SPSS Statistics 27, Armonk, New York, USA). Since the data were not normally distributed (Kolmogorov–Smirnov-test, *p* > 0.05 for all parameters), a non-parametric *U*-test (Wilcoxon) was performed to analyze possible effects of treatment (overall and for each group; pre- and post-treatment). Differences among groups for ordinal parameters (presence of microbial growth, cytology score, presence of endometritis, presence of clinical symptoms of endometritis) were analyzed using chi-square test, whereas differences among groups for the continuous variable (number of PMNs) were evaluated by performing the non-parametric Kruskal–Wallis test. For analysis of possible effects of cycle stage (before treatment and at the time of control examination), all described procedures were repeated. Significance was set at *p* < 0.05.

## 3. Results

One mare had to be excluded from the analysis due to missing microbiological examination results (control examination), resulting in a total number of *n* = 29 mares included in the analysis. The results of the initial examination, including reproductive status, age, breed, uterine microbiological and cytological assessment and biopsy scoring according to Kenney and Doig (26) and Schoon et al. (27) can be found in Table 1. Information on the cycle stages at the time of initial examination, treatment and control examination is provided in Table 2. None of the mares showed clinical signs of discomfort or systemic illness before, during or after treatment.

**Table 2:** Information on individual mare characteristics and cycle stage at the initial examination, the onset of treatment and control examination as determined by gynecological examination.

**Mare (no.)**	**Age (years)**	**Reproductive status of mare**	**Treatment group**	**Cycle stage (initial insemination)**	**Cycle stage (onset of treatment)**	**Cycle stage (control examination)**
1	20	Barren	Ozone	Diestrus	Diestrus	Diestrus
2	14	Maiden	Ozone	Estrus	Estrus	Diestrus
3	12	Barren	Ozone	Diestrus	Diestrus	Diestrus
4	6	Barren	Ozone	Estrus	Estrus	Diestrus
5	17	Maiden	Ozone	Estrus	Estrus	Estrus
6	6	Barren	Ozone	Estrus	Estrus	Diestrus
7	10	Barren	Ozone	Estrus	Estrus	Diestrus
8	15	Maiden	Ozone	Estrus	Diestrus	Diestrus
9	14	Foaling	Ozone	Estrus	Estrus	Diestrus
10	4	Maiden	TMS	Estrus	Estrus	Diestrus
11	8	Maiden	TMS	Estrus	Estrus	Diestrus
12	4	Maiden	TMS	Estrus	Estrus	Diestrus
13	18	Barren	TMS	Estrus	Estrus	Estrus
14	26	Barren	TMS	Diestrus	Diestrus	Estrus
15	10	Maiden	TMS	Estrus	Estrus	Diestrus
16	12	Barren	TMS	Estrus	Estrus	Diestrus
17	16	Barren	TMS	Estrus	Estrus	Diestrus
18	13	Foaling	TMS	Diestrus	Diestrus	Estrus
19	8	Barren	TMS	Diestrus	Diestrus	Diestrus
20	16	Barren	Air	Estrus	Estrus	Diestrus
21	5	Barren	Air	Diestrus	Diestrus	Estrus
22	9	Foaling	Air	Estrus	Estrus	Diestrus
23	11	Foaling	Air	Diestrus	Diestrus	Diestrus
24	14	Barren	Air	Estrus	Estrus	Estrus
25	19	Barren	Air	Diestrus	Diestrus	Diestrus
26	16	Maiden	Air	Diestrus	Diestrus	Estrus
27	23	Barren	Air	Estrus	Estrus	Estrus
28	12	Maiden	Air	Diestrus	Diestrus	Diestrus
29	Unknown	Barren	Air	Diestrus	Diestrus	Estrus

Gynecological examination revealed no differences among groups for signs of endometritis (e.g., endometrial hyperedema, intraluminal fluid or vaginal discharge) before and after treatment, although there was a tendency that more mares (4/10) depicted signs of endometritis after treatment with air as compared to ozone (2/9) and TMS (0/10, *p* = 0.087).

**Figure 2:**
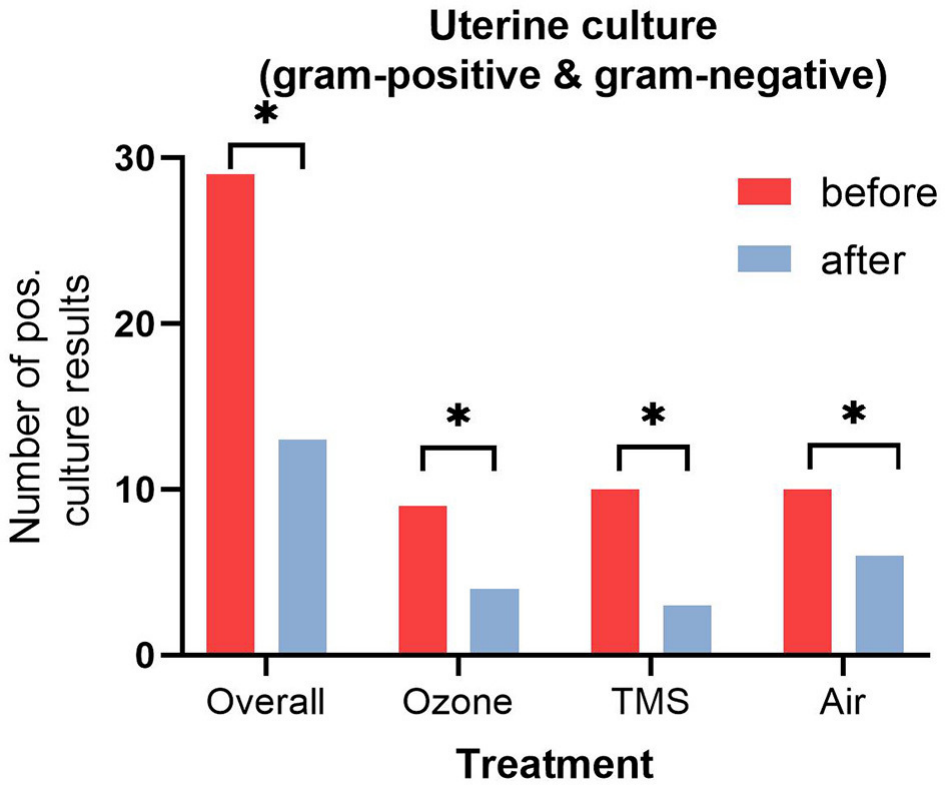
Number of positive uterine culture results (gram-positive and gram-negative bacteria) before and after treatment. Asteriks (^*^) indicate significant difference between pre- and post-treatment results as determined by non-parametric *U*-test (Wilcoxon; *p* < 0.05).

**Figure 3:**
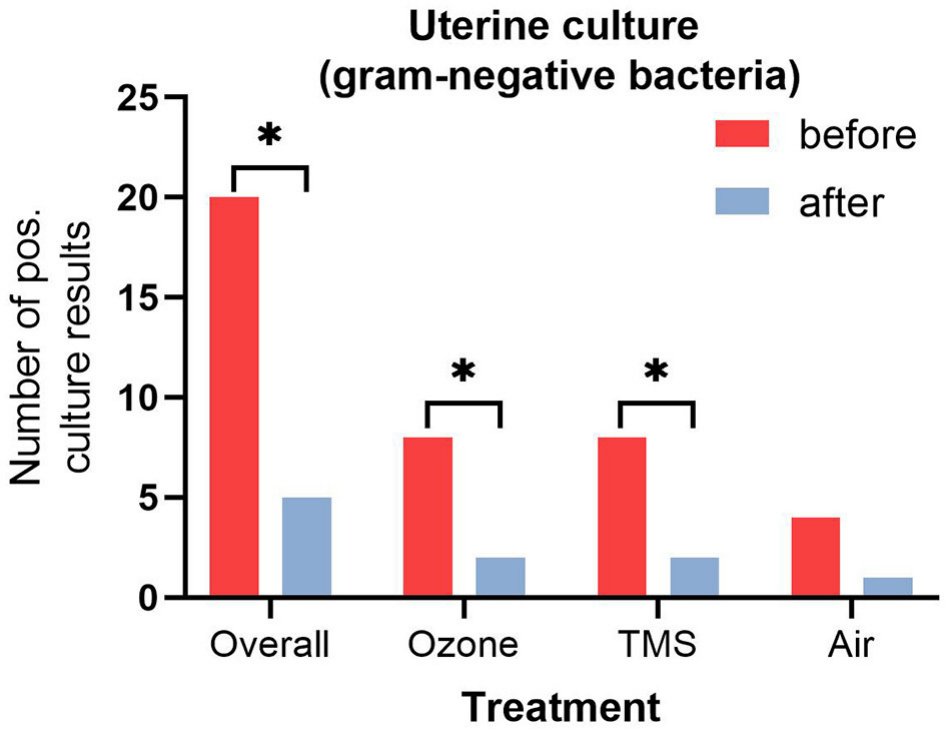
Number of positive uterine culture results (gram-negative bacteria) before and after treatment. Asteriks (^*^) indicate significant difference between pre- and post-treatment results as determined by non-parametric U-test (Wilcoxon; *p* < 0.05).

No differences for microbiological results were found between groups after their respective treatment, but an overall effect of treatment was observed (Figure 2, *p* < 0.05). All isolated bacteria were sensitive to TMS and did not show unexpected antibiotic susceptibility results. Less gram-negative bacteria (number of positive cultures) were found after treatment with ozone and TMS but not after treatment with air (Figure 3). For gram-positive bacteria (number of positive cultures), no treatment effects were noted for ozone and air (*p* > 0.05), but for TMS (*p* < 0.05; data not shown). The comparison of effects among treatments on growth of gram-positive and gram-negative bacteria revealed no statistically significant differences (*p* > 0.05).

**Figure 4:**
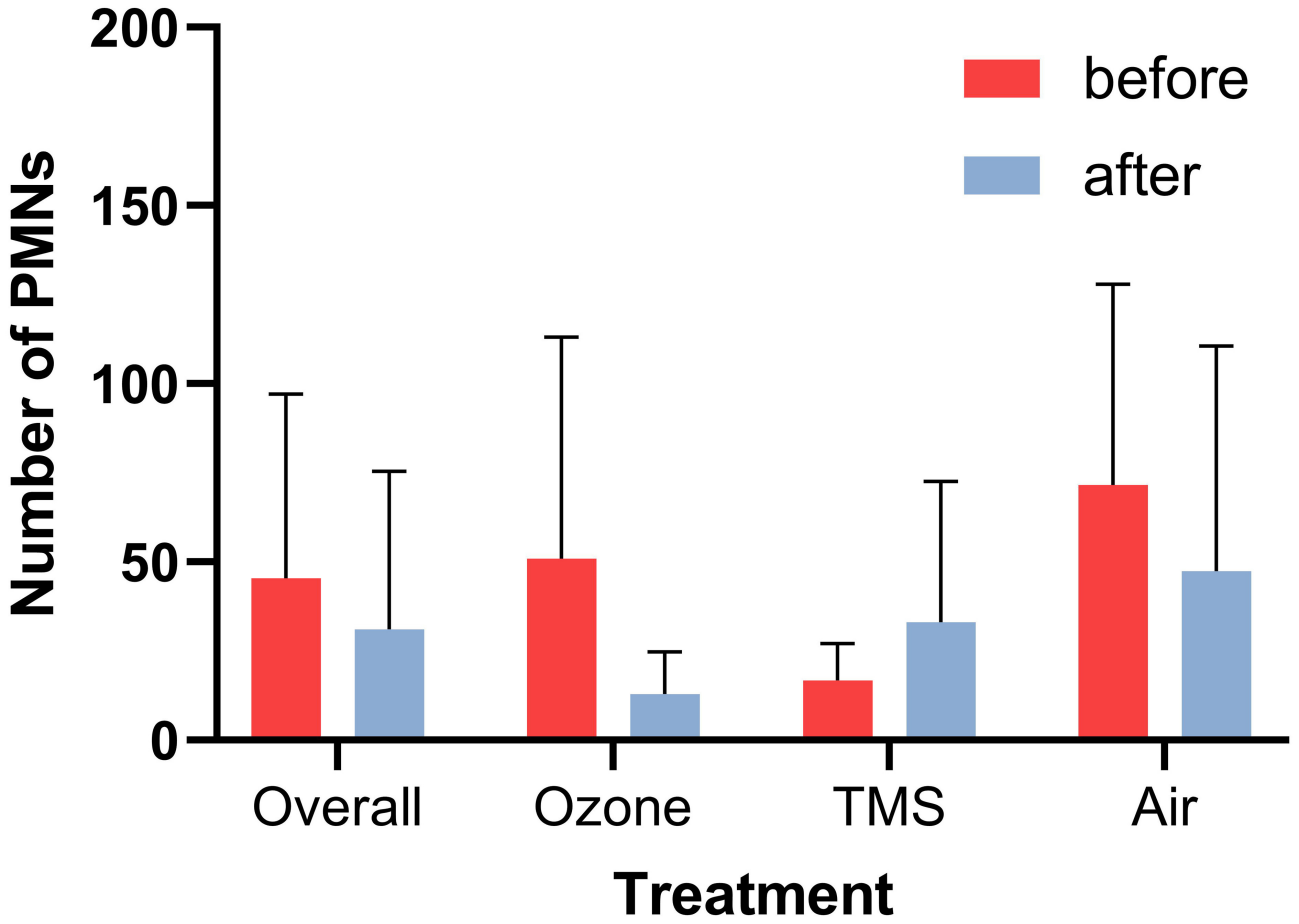
Number of polymorphnuclear leukocytes (PMNs) per 10 high power fields before and after treatment as determined *via* endometrial cytology (mean + SD). No statistically differences were determined (*p* > 0.05).

No effect of cycle stage on the results was observed, except for the number of PMNs. At the time of the control examination, diestrous mares had lower numbers of PMNs as compared to estrous mares (Kruskal–Wallis: *p* < 0.05; data not shown). Neither an effect of treatment nor treatment group on the number of PMNs (*p* > 0.05; Figure 4) and cytology score was noted (*p* > 0.05; data not shown).

**Figure 5:**
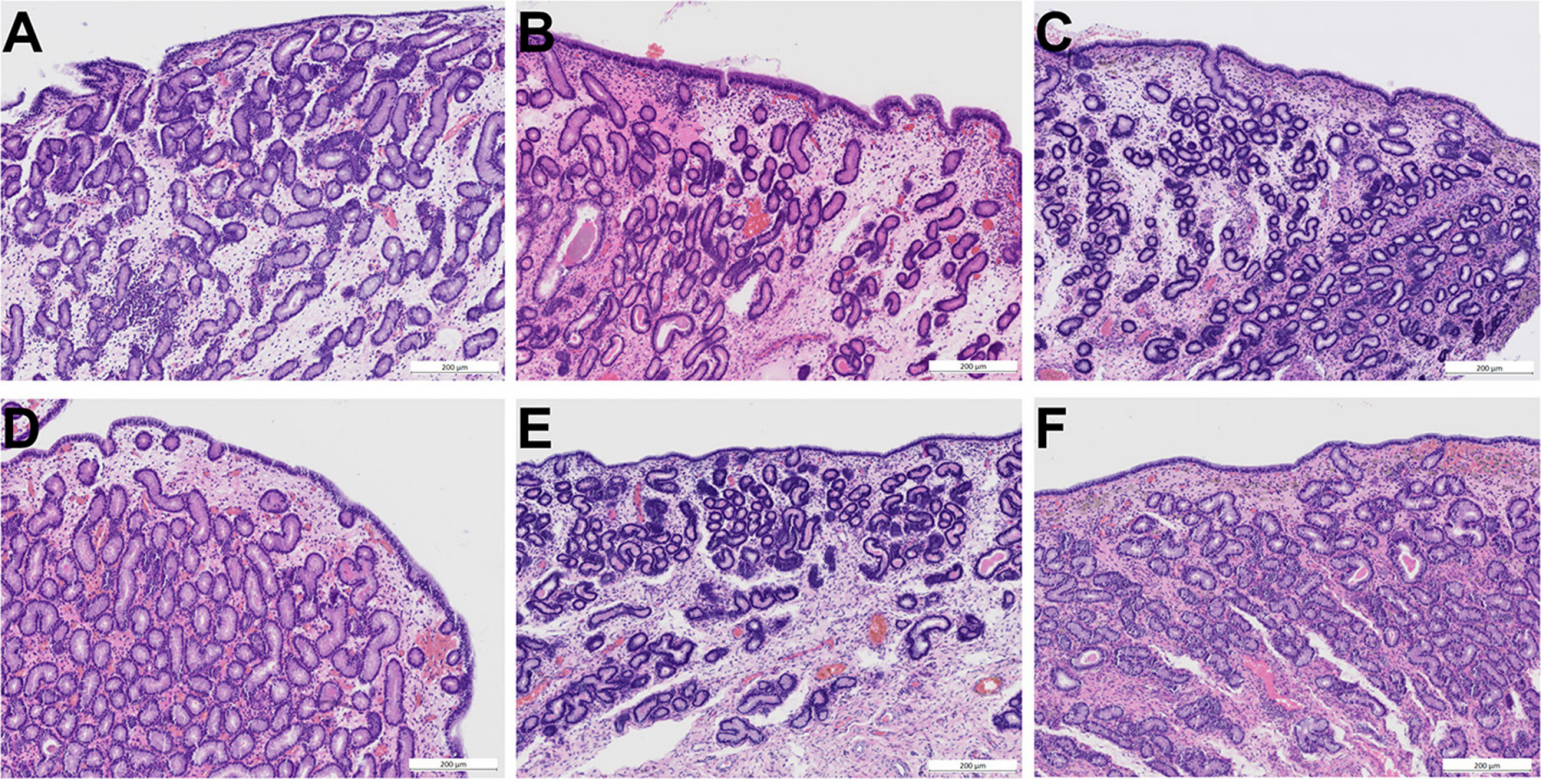
Photomicrographs (HE staining) of endometrial biopsies obtained before treatment **(A–C)** and after treatment **(D–F)**. **(A)** Endometrium of mare 3 before treatment with ozone (age 12, category III, moderate signs of endometritis). **(B)** Endometrium of mare 15 before treatment with trimethoprim-sulfadimethoxine (age 10, category III, moderate signs of endometritis). **(C)** Endometrium of mare 22 before treatment with air (age 9, category III, mild signs of endometritis). **(D)** Endometrium of mare 3 after intrauterine insufflation of ozone showing mild signs of endometritis and an intact surface epithelium. **(E)** Endometrium of mare 15 after systemic treatment with trimethoprim-sulfadimethoxine showing mild signs of endometritis and an intact surface epithelium. **(F)** Endometrium of mare 22 after intrauterine insufflation of air showing mild signs of endometritis and an intact surface epithelium. Scale bars = 200 μm. All categories according to Kenney and Doig (26), modified by Schoon et al. (27).

Pathohistological examination of biopsies revealed neither differences between groups nor before and after treatment with regards to presence of inflammatory cells, fibrosis, epithelial desquamation as well as extravascular erythrocytes (Figure 5).

## 4. Discussion

Since endometritis is one of the major problems encountered in equine medicine (30), a lot of research has been performed on diagnostic and treatment strategies of this condition (6). In the present study performed in a clinical setting, effects of three different treatments (intrauterine ozone administration, intrauterine air insufflation, and systemic administration of trimethoprim-sulfadimethoxine) on microbial growth, cytological findings, and endometrial histomorphology were analyzed and compared. The treatment of bacterial endometritis usually involves antibiotics, which can either be administered locally or systemically. Since there is still conflicting evidence concerning the administration route (31), one aim of the present study was to evaluate the efficacy of trimethoprim-sulfadimethoxine in a clinical setting.

Due to an increasing prevalence of antimicrobial-resistant uterine pathogens (32–34) and the lack of adequate novel antibiotic drugs in equine medicine, non-traditional treatment options have gained interest in the past years (15). Among others, intrauterine administration of ozone has been reported as a valuable treatment option by practitioners. Until now, there is only one study evaluating the efficacy of insufflation of ozone into the uterus in mares, diagnosed with bacterial endometritis (24). The authors found an ozone-air-mixture (40 μg/ml for 10 min) to be efficient in reducing intrauterine bacterial growth in 89% of the mares. Furthermore, the authors reported a decrease in the number of PMNs as determined by cytological examination. In the present study, two insufflations of an ozone-air-mixture with a higher concentration of ozone (80 μg/ml) were also efficient in reducing bacterial growth but did not result in a significantly lower number of PMNs. The different results may be attributed to differing study designs: In the present study, the control examination was performed seven days after the last treatment irrespective of the cycle stage as often done by practitioners (11), whereas Ávila et al. (24) obtained samples during the subsequent estrus, probably resulting in a better comparability of the cytological findings. At the same time, this method bears the risk of missing immediate treatment effects. Another recent study investigated possible effects of uterine lavage using ozonated saline in mares on the number of PMNs, endometrial architecture and pregnancy rate. In accordance with the present study, no effects of the ozonated saline on the number of PMNs and the endometrial architecture were observed. Moreover, mares (*n* = 10) treated with ozonated saline 4 h after insemination depicted high pregnancy rates (9/10 mares) (35).

For prevention of possible leakage of gas, using an embryo flushing catheter would have been an interesting different approach. However, the use of an insemination pipette in combination with the manual occlusion of the cervix prevented gas leakage into the vaginal cavity effectively and provided an easy-to-use method for the application of the ozone-air-mixture. Additionally, Ávila et al. (24) did not report transcervical leakage of gas using the same method as described in the present study.

Regarding microbiology, all treatments resulted in reduced intrauterine microbial growth. Surprisingly, this was even true for insufflation of air into the uterus, resulting in negative microbiology results in 4/10 mares. In this case, it is surely debatable whether a control group without any manipulation of the uterus would have been a more appropriate control treatment than the insufflation of air. However, there is only little knowledge about the self-healing capability of mares concerning bacterial endometritis. A study published in 1969 investigated the elimination of experimentally introduced β-hemolytic *Streptococcus* and *Pseudomonas* organisms into the uteri of five mares (two barren and three maiden mares) and found out that all mares were able to eliminate the bacteria from the uterus within 4–15 days (36). Here, an effect of cycle stage would have been conceivable, but could not be determined while analyzing the effect of cycle stage on the treatment outcome. Although mares in the study were infected experimentally and mares in the present study were naturally infected, the capability of mares to eliminate infectious pathogenic organisms from the uterus should be kept in mind when designing experiments on treatment efficacy. Interestingly, a study from Kentucky demonstrated that healthy stallions, harboring potentially pathogenic bacteria on their external genitalia, did not infect more mares when breeding by live cover as compared to healthy stallions without a pathogenic genital microflora (37). Although this finding cannot be attributed to the self-healing capability of mares in terms of bacterial endometritis, it demonstrates the natural immunity of mares against some pathogenic bacteria as it has been postulated only recently in cattle (38).

Systemic antibiotic treatment of chronic infectious endometritis is favored by most practitioners in Germany and most veterinarians employ trimethoprim-sulfadimethoxine as a first-line antibiotic drug for treatment of the disease (11). Regarding the efficacy of systemic treatment, a study by Davolli et al. (14) demonstrated that the potentiated sulfonamide reaches tissue concentrations above the minimum inhibitory concentration (MIC) of *S. equi* ssp. *zooepidemicus* and *E. coli* when administered orally at a dosage of 24 mg/kg twice daily. The authors concluded that oral treatment should be an efficient and viable treatment for bacterial endometritis (14). Although this treatment option is extensively used in the field, data on the expectable success rate from clinical cases was lacking. Here, we demonstrated elimination of microbial growth in 7/10 mares with intrauterine growth of pathogenic bacteria susceptible to trimethoprim-sulfadimethoxine after systemic application of trimethoprim-sulfadimethoxine (30 mg/kg, twice daily for 5 days perorally). Since clinical cases were used and some mares were positive for gram-positive as well as gram-negative pathogens, no clear statement about the efficacy of this treatment against either gram-positive or gram-negative bacteria can be made. Given the fact, that all bacteria were susceptible to the drug *in vitro* and required dosage was met, the clinical success rate was expected to be higher. In the context of stricter legislation on the use of antimicrobials in animal husbandry and in order to improve clinical evidence of various treatment methods, more data on clinical success rates is needed. A possible confounding effect on the efficacy of antibiotic drugs *in utero* might result from bacterial biofilm formation (6, 31). However, the ability to produce biofilms has not yet been demonstrated for ß-hemolytic streptococci, but only for *E. coli, Pseudomonas aeruginosa* and *Klebsiella pneumonia* in the context of equine bacterial endometritis (39, 40). Here, the capability of the isolated bacteria to form biofilms was not assessed. Therefore, an effect of possible biofilms caused by *E. coli* on the efficacy of the antibiotic or ozone treatment cannot be ruled out. Generally, additional therapies should be administered whenever biofilm formation is suspected [for review: (6, 15)].

While clinical examinations revealed that none of the treatments affected general health and condition of mares neither negatively nor positively, there were also no negative effects of the treatments on the genital health as determined by gynecological examination.

In addition to gynecological examination, cytological examination was performed to assess the inflammatory reaction of the endometrium after intrauterine and systemic treatment. However, cytological examination revealed neither significant changes of the number of PMNs nor cytology scoring after treatment. Furthermore, no differences among treatments were noticed. Interestingly, there are no reports on improvement of cytology scoring in mares after therapy of endometritis. Intrauterine treatments and manipulations may also be a cause for the insignificant changes in the number of PMNs in the present study (6). However, the presence of some PMNs can be considered normal to a certain degree as PMNs were also found in cytological samples from clinically healthy mares during estrus (41, 42). Moreover, effects of cycle stage on cytology were observed and have to be considered while interpreting the results, since cytological samples were not only obtained during estrus but also during diestrus. Here, a more consistent sampling protocol, regarding cycle stage as well as sampling method [as performed by Ávila et al. (24)], could have been beneficial but, unfortunately, could not be integrated in sampling protocol in the clinical practice.

Regarding the endometrial biopsies, considered to be the gold standard for diagnosis of endometritis in mares (41, 43), signs of endometritis (e.g., presence of PMNs or other leukocytes) could not be found in all biopsies, obtained during initial examination of the mares. No PMNs were present in 8/29 biopsies, although cytological and microbiological assessment was consistent with presence of endometritis in these mares. Again, the presence of some PMNs can be considered physiological especially during estrus and in clinically healthy mares (44, 45). Since no effect of treatment was observed histologically, it can be concluded that none of the therapies impairs the functionality of the endometrium. However, there was no improvement regarding the presence of endometritis after any treatment. This result is in line with other studies investigating effects of intrauterine endometritis treatment in horses. A study by McDonnell and Watson (46) showed presence of mild to moderate endometritis 4 days after intrauterine ampicillin treatment. Moreover, Melkus et al. (47) even observed an increase of endometrial PMNs after treatment (24 and 72 h) with 0.9 % saline solution. Although both studies reported a shorter treatment-to-biopsy-interval, they support the hypothesis that every manipulation of the uterus (e.g., insufflation of air during hysteroscopy, lavage with ozonated saline) bears the risk of a local inflammatory reaction (35, 48, 49). Additionally, most mares were categorized as grade IIb or III (26/29), demonstrating chronic alterations of the endometrium, possibly interfering with the uterine clearance of the mares. Thus, the endometrial conditions have to be taken into account while interpreting the results, especially the lacking treatment effects on the number of PMNs. Due to the chronic nature of these alterations, no effect of treatment on the presence of endometrosis was noted. According to the authors' knowledge, no information on effects of systemic treatment of endometritis exist but would be beneficial for comparison of potential impacts of the application route on the endometrial histomorphology.

Moreover, there were neither negative nor positive effects of the cycle stage during the respective treatments gynecological, microbiological, cytological and histopathological examination results. Normally, systemic antibiotic treatment is performed at any cycle stage, while intrauterine antibiotic treatment should be performed during estrus (3). Here, there were no negative effects of intrauterine insufflation on uterine health observed, although intrauterine treatments were performed in 9/19 mares during diestrus (3/9 mares in the ozone group and 6/10 mares in the air group). Due to the small group sizes, no final conclusion on the efficacy and safety of intrauterine ozone insufflation during diestrus can be made. Besides the small group size, the high proportion of maiden mares, presented for insemination, with bacterial endometritis has to be discussed as well, since false positive culture and cytology results could have impaired the results here. Nevertheless, the majority of maiden mares (7/9) were older than 5 years and presence of PMNs was revealed by cytology in six of nine mares. Three mares without cytological diagnosis of endometritis had a positive culture result and endometritis was confirmed *via* histopathology. Intrauterine growth of pathogenic bacteria can also be found in young (< 5 years old) and in older maiden mares (2, 50). Therefore, the authors consider the risk of mares, false positive for bacterial endometritis, to be low.

In conclusion, the fact that the present study has been performed in a clinical setting resulted in some limitations (e.g., no cross-over design, lacking additional oral placebo treatment group, treatment and sample collection irrespective of the mares' cycle stage and a rather small number of mares), which are typical for studies performed in the field. Nevertheless, performing the study in a clinical setting might reflect the situation in routine practice more closely. Therefore, the applicability of the results under actual field conditions may be supported. Summarizing the results, trimethoprim-sulfadimethoxine and intrauterine ozone insufflation are clinically safe treatment options for bacterial endometritis in mares as determined by cytological and histopathological examination. The efficacy of both treatments in reducing bacterial growth did neither result in a complete absence of intrauterine bacterial growth nor reduction of positive uterine cultures compared to local air insufflation. Therefore, more studies should be performed to address the potential of ozone therapy to treat uterine infections in mares.

## Data availability statement

The raw data supporting the conclusions of this article will be made available by the authors, without undue reservation.

## Ethics statement

The animal study was reviewed and approved by Lower Saxony State Office for Consumer Protection and Food Safety (33.8-42502-04-20/3565). Written informed consent was obtained from the owners for the participation of their animals in this study.

## Author contributions

MK, LH, DB, AG, RU, and HS participated in the design of the study. MK, LH, DB, AH, and AT collected the data and performed the statistical analyses. MK, DB, and HS drafted the manuscript. All authors read and approved the final manuscript.
